# Efficacy Analysis of Double-Low Dynamic Contrast-Enhanced CT and Hepatic Extracellular Volume Fraction in the Diagnosis of Liver Fibrosis

**DOI:** 10.1155/2022/8089914

**Published:** 2022-08-17

**Authors:** Zhandong Liang, Yanxia Liu, Yuanwen Nie

**Affiliations:** ^1^Department of Medical Imaging, The First Hospital Affiliated of Hebei North University, Zhangjiakou 075000, Hebei, China; ^2^Department of Ultrasound, Zhangjiakou Infectious Diseases Hospital, Zhangjiakou 075000, Hebei, China; ^3^Department of Hepatobiliary Surgery, The Second Affiliated Hospital of Mudanjiang Medical University, Mudanjiang 157000, Heilongjiang, China

## Abstract

**Objective:**

The aim of the study was to analyze the efficacy of double-low dynamic contrast-enhanced CT (DCE-CT) and hepatic extracellular volume fraction (fECV) in the diagnosis of liver fibrosis (LF).

**Methods:**

A total of 200 patients with LF and cirrhosis who underwent the histopathological examination of liver biopsy and multiphase DCE-CT of the liver at the same time in our hospital (January 2020–December 2020) were selected as the research subjects, and the degree of liver fibrosis was staged according to pathological criteria to analyze the clinical diagnostic value of double-low DCE-CT and fECV.

**Results:**

Compared with the S2–S4 group, the S1 group had obviously higher Eaorta and HCT values (*P* < 0.05), a lower fECV value (*P* < 0.001), and lower serum IVC and LN levels (*P* < 0.001). Serum IVC and LN levels were positively correlated with fECV (*r*_1_ = 0.803 and *r*_2_ = 0.890; *P* < 0.001). The fECV had the highest specificity and negative predictive value in the diagnosis of S1 and had the highest sensitivity and positive predictive value in the diagnosis of S2–S4.

**Conclusion:**

The double-low DCE-CT and fECV can provide a reliable basis for the clinical diagnosis of LF, and their results will provide a new direction for the treatment of LF and have a high application value in the clinical practice.

## 1. Introduction

Liver fibrosis (LF), the abnormal accumulation of liver extracellular matrix, especially collagen fibers [1], with the clinical symptoms of dyspepsia, anorexia, and fatigue, can progress to cirrhosis or liver cancer without timely clinical intervention. It has been found that LF is a compensatory repair reaction after liver injury and a process of dynamic pathological changes [2]. Since the occurrence of LF runs through the pathological process of chronic liver diseases, it is speculated that effectively preventing or delaying the occurrence of LF is beneficial to the treatment of chronic liver diseases. Therefore, early diagnosis and treatment are of great significance to restore the function and structure of damaged liver tissues. The histopathological examination results of liver biopsy are taken as the gold standard for the diagnosis of LF, but this invasive examination method with too little sampling can easily cause complications such as pneumothorax and local pain of puncture, resulting in its limited clinical application [3, 4]. In recent years, noninvasive diagnostic techniques have been widely used to evaluate LF. Double-low dynamic contrast-enhanced CT (DCE-CT) obtains the CT images of the routine phase with a low contrast medium concentration and low radiation dose, which can not only greatly improve the accuracy of evaluating liver diseases but also meet the needs of clinical application [5, 6]. The application of this imaging technology can lessen the interference of noise on images, reduce artifacts, achieve an easier and more accurate reading of images, and facilitate better disease diagnosis by physicians. Hepatic extracellular volume fraction (fECV) can measure the extracellular space as the percentage of noncellular tissue volume. It has certain advantages in clinical diagnosis because this value is a physiologically intuitive measurement unit independent of the field strength and may be more suitable for follow-up investigation and study with different imaging methods [7, 8]. In this study, the histopathological examination results of liver biopsy of the selected subjects were taken as the gold standard, and the clinical efficacy of fECV for the diagnosis of LF was investigated by quantitative analysis of liver extracellular matrix volume of double-low DCE-CT. This study aimed to provide new technologies and ideas for the diagnosis and treatment of LF, better guide clinicians to formulate and adjust treatment plans in time, improve disease prognosis, and enhance the quality of life of patients.

## 2. Materials and Methods

### 2.1. General Information

**Figure 1:**
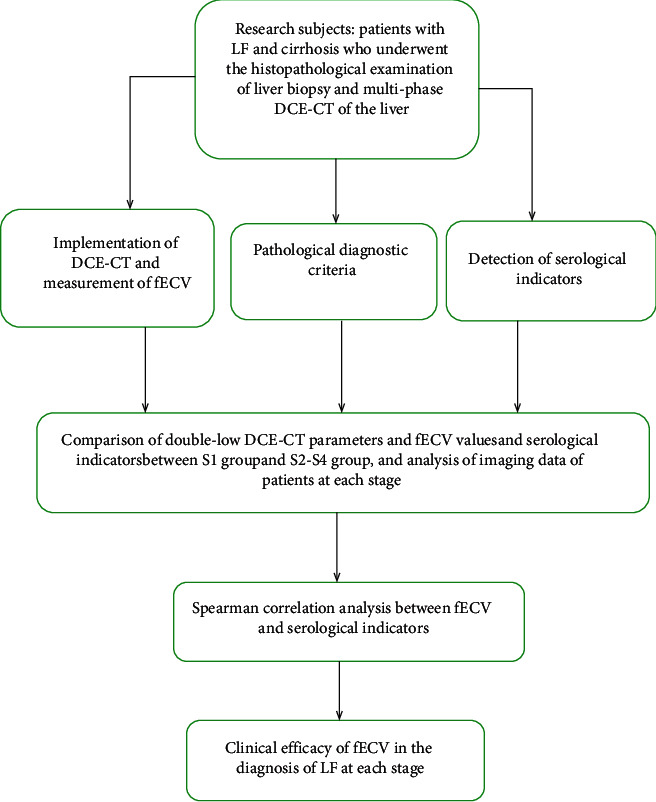
Flow diagram of the study.

A total of 200 patients with LF and cirrhosis who underwent the histopathological examination of liver biopsy and multiphase DCE-CT of the liver at the same time in our hospital (January 2020–December 2020) were selected as the research subjects, including 115 males and 85 females aged 24–63 years, with an average age of (41.97 ± 12.11) years and an average BMI of (21.87 ± 1.59) kg/m^2^. In terms of the education degree, there were 28 undergraduates, 32 junior college students, 52 high school students, 56 junior school students, 21 primary school students, and 11 illiterate people, of whom 96 patients lived in urban areas and 104 in rural areas. This study was approved by the ethics committee of our hospital and conformed to the Declaration of Helsinki (2013) [9]. The flow diagram of the study is shown in Figure 1.

### 2.2. Inclusion Criteria and Exclusion Criteria

  Inclusion criteria were as follows: (1) patients had no previous clinical treatment and were aged over 18 years; (2) patients and their families knew the purpose of the experiment and signed the consent form.
  Exclusion criteria were as follows: (1) patients with mental disorders; (2) patients with advanced cirrhosis and liver tumors; (3) pregnant or lactating women.

### 2.3. Methods

#### 2.3.1. Double-Low DCE-CT

The 64-slice spiral CT scanner (manufacturer: TOSHIBA company; model: Aquilion ONE) was used for scanning. After fasting for more than 4h before scanning, the patients were instructed to take the supine position, and the scanning range was from the diaphragmatic dome to the lower edge of the liver and spleen. After plain scanning, a high-pressure syringe (OptiVantage DH) was used for the intravenous bolus injection of 60 mL of the contrast agent iodixanol (NMPA approval no. H20113465; manufacturer: Beijing Beilu Pharmaceutical Co., Ltd.; specification: 270 mgI/mL) at a rate of 4.0 mL/s, followed by the injection of normal saline (30 mL) at the same speed. The multiphase-enhanced scanning time was about 25 s in the arterial phase, 60 s in the portal phase, 180 s in the equilibrium phase, and 600 s in the delayed phase. The scan parameters of patients were 80 KV, and automatic milliampere (range of 9–500 mA) was combined with adaptive iterative dose reduction (AIDR) technology. The volume data of each phase were imported into Vitrea fx workstation for image analysis and processing.

#### 2.3.2. Measurement of fECV

With the division method of five lobes and eight segments in the liver, the ROI of about 1.0 cm^2^ in each segment was selected to measure the CT values of the segments in plain scanning, equilibrium phase, and delay phase. Upon the selection of ROI, the blood vessels, bile duct, and occupying lesions were avoided. The CT values of the abdominal aorta in plain scanning, equilibrium phase, and delayed phase were also measured, and the ROI was as large as possible while avoiding the vascular wall. All the above measurements were performed three times, and the average values were taken. Absolute enhancement values of liver parenchyma (Eliver) and aorta (Eaorta) of each segment = CT values in the equilibrium phase (delayed phase)—CT values in the plain phase. In equilibrium, the distribution volume of the contrast medium was 1-hematocrit (1-Hct). fECV (%) = Eliver × [100 − Hct (%)]/Eaorta.

### 2.4. Pathological Diagnostic Criteria

S1 referred to the fibrous enlargement of the portal area and localized perisinusoid fibrosis. S2 referred to the fibrosis around the portal area, formation of fibrous septa in the lobule, and retention of lobule structure. S3 showed a large number of fibrous septa, with lobular structural disorders and no cirrhosis. S4 showed evident structural disorders of the lobule, with early cirrhosis.

### 2.5. Detection of Serological Indicators

Fasting venous blood (3 mL) was collected on the day of imaging examination, and the supernatant was collected after routine centrifugation for detection. Serum IV collagen (IVC) and laminin (LN) were detected by radioimmunoassay, with the IVC kits purchased from Shenzhen New Industries Biomedical Engineering Co., Ltd. and LN kits from Shanghai Xinfan Biotechnology Co., Ltd. The detection was performed strictly according to the kit instructions.

### 2.6. Statistical Methods

The quantitative data were expressed as mean ± standard deviation ($\bar{x}$ ± *s*), and the parameter or nonparameter tests were adopted to analyze the comparison among multiple groups according to whether the data were normally distributed and homogeneity of variance. One-way ANOVA was used to analyze the statistical differences in the fECV values between groups and within groups, and Spearman rank correlation was used to analyze whether there was a correlation between fECV values and cirrhosis staging. The receiver operating characteristic (ROC) curves were used to analyze the accuracy of fECV in the diagnosis of LF and define the demarcation values. With the histopathological examination results of liver biopsy as the gold standard, sensitivity (Se) and specificity (Sp) of fECV values were calculated. The area under the curve (AUC) between 0.5 and 0.7 indicated a low diagnostic value, that between 0.7 and 0.9 suggested a moderate diagnostic, and that above 0.9 showed a high diagnostic value. All statistical analysis was performed using SPSS19.0 software, and the differences were statistically significant at *P* < 0.05.

## 3. Results

### 3.1. Histopathological Diagnosis of Liver Biopsy

Among the 200 patients, 42 cases were diagnosed as stage S1, 47 cases as stage S2, 56 cases as stage S3, and 55 cases as stage S4 after the histopathological examination of liver biopsy. According to the severity of LF, the patients were split into the S1 group (*n* = 42) and the S2–S4 group (significant liver fibrosis, *n* = 158).

### 3.2. Comparison of Double-Low DCE-CT Parameters and fECV Values

Table 1 shows obviously higher Eaorta and HCT values (*P* < 0.05) and a lower fECV value (*P* < 0.001) in the S1 group than in the S2–S4 group.

### 3.3. Comparison of Serological Indicators

The serum IVC and LN levels were notably lower in the S1 group than in the S2–S4 group (*P* < 0.001), as presented in Table 2.

### 3.4. Imaging Data of Different LF Stages

**Figure 2:**
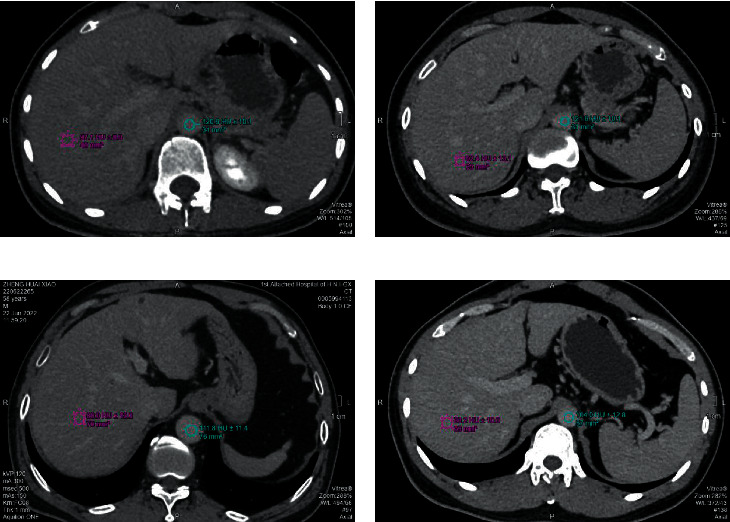
Note: (a) an image of S1; (b) an image of S2; (c) an image of S3; (d) an image of S4.

Imaging data of different LF stages are shown in Figure 2.

### 3.5. Spearman Correlation Analysis between fECV and Serological Indicators

**Figure 3:**
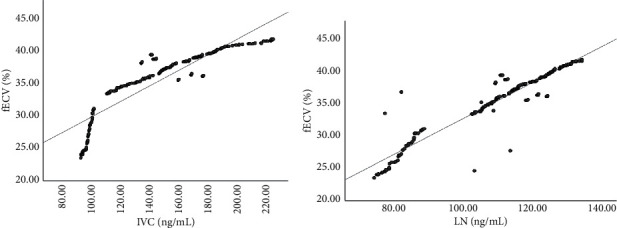
Spearman correlation analysis between fECV and serological indicators. Note: (a) correlation analysis between the IVC level and fECV and (b) correlation analysis between the LN level and fECV.

The Spearman rank correlation analysis showed that the serum IVC and LN levels were positively correlated with fECV (*r*_1_ = 0.803 and *r*_2_ = 0.890; *P* < 0.001) (See Figure 3).

### 3.6. Clinical Efficacy of fECV in the Diagnosis of LF at Each Stage

**Figure 4:**
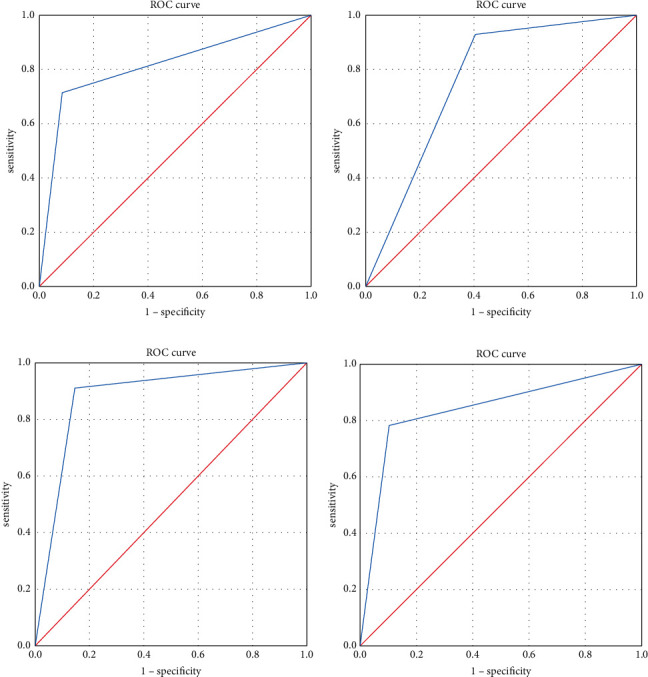
ROC curves of fECV for LF diagnosis at each stage. Note: (a) ROC curve of S1 diagnosis; (b) ROC curve of S2–4 diagnosis; (c) ROC curve of S3-4 diagnosis; (d) ROC curve of S4 diagnosis.

The fECV had the highest specificity and negative predictive value in the diagnosis of S1 and had the highest sensitivity and positive predictive value in the diagnosis of S2–S4. Details are presented in Table 3 and Figure 4.

## 4. Discussion

Liver fibrosis (LF), a compensatory repair reaction after liver injury and a process of dynamic pathological changes, can cause cirrhosis or liver failure with disease progression, increasing the prevalence of liver malignant tumors [10, 11]. A clinical study [12] has shown that early diagnosis and treatment are of great significance to restore the structure and function of damaged liver tissues due to the potential reversibility of LF and cirrhosis. The pathological examination of liver biopsy has many disadvantages such as pneumothorax and puncture pain, resulting in poor clinical compliance [13, 14]. The extensive clinical application of contrast-enhanced CT diagnostic techniques enables the common use of contrast agents, followed by wide attention of physicians on the adverse reactions of high radiation and contrast agents. High radiation doses will cause long-term adverse effects on high-sensitive organs such as the thyroid, thereby increasing the risk of iatrogenic renal failure. With the advantages of low dose and low radiation, the double-low DCE-CT technology not only effectively reduces the damage of the high-dose X-ray radiation caused by scanning but also markedly reduces the risk of contrast-induced nephropathy caused by the injection of high-dose contrast agents [15], which has been confirmed in diseases such as pulmonary embolism and solitary pulmonary nodules [16, 17]. In this study, the double-low DCE-CT diagnostic technique could not only obtain the perfusion information of the lesions but also comprehensively evaluate the overall morphology and blood supply of the liver lesions. At the same time, double-low DCE-CT could obtain the CT images of the routine phase through the AIDR technology, with the image quality qualified for imaging diagnosis. Meanwhile, the implementation of CT scanning could also obtain the quantitative analysis of LF while detecting liver cancer or localized lesions of the liver, which enabled easier calculation of the fECV values without a lot of postprocessing time [18–20]. The combination of low-dose imaging, morphology, and molecular imaging provides a broad space for the accurate diagnosis of LF and cirrhosis.

Based on the previous theoretical research, the patients with LF and cirrhosis who underwent liver biopsy and multiphase DCE-CT of the liver at the same time in our hospital were selected as the research subjects in this study to explore the clinical application advantages of the study protocol through the detection of CT parameters and fECV values and bring the best scheme for patients. This study showed obvious differences in the fECV values measured by CT among patients at different LF stages and increased fECV values with the aggravation of the disease. In addition, fECV has a relatively high diagnostic accuracy for different LF stages. Compared with the plain CT scan, the contrast agents of the double-low DCE-CT scan after injection can freely enter and exit the blood vessels and cells [21] and finally reach a state of equilibrium. In addition, using the obtained fECV to evaluate LF is of application value for patients with contraindications to ultrasound or MRI [20], and CT determination of fECV can cover the whole liver (including the left lobe of the liver) of the subject [22], which greatly increases the accuracy of clinical diagnosis. The experimental results are better than those of Wu et al. [23]. In this study, the internal lesions of patients at each stage were better observed with the LF imaging data, so as to improve the accuracy of clinical diagnosis of the disease. At the same time, with good visual effects, the imaging technology not only obtains the perfusion information of the lesions but also comprehensively evaluates the whole morphology and blood vessels of the liver lesions, which greatly meets the needs of clinical diagnosis. This study also found that fECV did not have the highest accuracy and specificity in the diagnosis of stage S2–4, so patients at this stage are suggested to be evaluated with the addition of other methods in order to achieve more ideal diagnostic efficacy. Many studies have shown that CT has obvious advantages in the diagnosis and evaluation of early LF, which will facilitate early clinical intervention to reduce the incidence of liver cirrhosis and liver cancer [24, 25]. The main contribution of this study lies in that the double-low DCE-CT and fECV can facilitate a better diagnosis of LF, which is a breakthrough in traditional diagnostic techniques and has extensive guiding significance in the diagnosis of liver diseases. This undoubtedly will become the development direction of future medicine. Of course, this study also has some shortcomings, such as the unevenly distributed number of cases in each stage and the small sample size. In addition, this study only investigates the serum IVC and LN levels of patients, lacking representativeness, which affects the results to some extent. Although continuous progress has been made in modern medical diagnosis and treatment technology, there are still many difficulties in the diagnosis of LF. Therefore, more efforts should be made to explore more efficient diagnostic techniques, optimize the current diagnostic modes, and provide more reliable data for the diagnosis of LF.

In conclusion, the quantitative analysis of the liver extracellular matrix volume of double-low DCE-CT is a promising research field, and its development further improves the accuracy and specificity of the diagnosis of liver diseases, which also provides a reliable basis for clinicians to formulate and adjust treatment plans in a timely manner.

## Figures and Tables

**Table 1:** Comparison of CT parameters and fECV values ($\bar{x}$ ± *s*).

Group	*N*	Eliver (HU)	Eaorta (HU)	HCT (%)	fECV (%)
S1 group	42	25.69 ± 5.17	44.19 ± 5.61	41.14 ± 2.88	27.04 ± 2.46
S2–S4 group	158	24.77 ± 5.34	41.06 ± 4.67	36.96 ± 3.27	37.86 ± 2.38
*T*		0.999	3.695	7.540	26.004
*P*		0.319	<0.05	<0.001	<0.001

**Table 2:** Comparison of serological indicators ($\bar{x}$ ± *s*, ng/mL).

Group	*N*	IVC	LN
S1 group	42	96.99 ± 2.73	81.42 ± 4.20
S2–S4 group	158	160.63 ± 29.96	117.90 ± 8.76
*T*		13.726	26.164
*P*		<0.001	<0.001

**Table 3:** Clinical efficacy of fECV in the diagnosis of LF in each stage.

Staging	AUC	Se (%)	Sp (%)	Positive predictive value (%)	Negative predictive value (%)	95%CI
S1	0.816	64.62	92.94	77.78	92.40	0.731–0.901
S2–4	0.763	90.29	79.25	93.49	71.19	0.668–0.858
S3-4	0.882	89.52	89.90	91.74	87.25	0.829–0.935
S4	0.839	78.57	92.36	82.09	90.63	0.769–0.909

## Data Availability

The data that support the findings of this study are available on reasonable request from the corresponding author.
